# Evaluation of vision transformers for the detection of fullness of garbage bins for efficient waste management

**DOI:** 10.3389/frai.2025.1612080

**Published:** 2025-09-09

**Authors:** Parakram Singh Tanwer, Shishir Maheshwari, Sushree Behera, Amit Chauhan, T. Sunil Kumar

**Affiliations:** ^1^Department of Data Science and Artificial Intelligence, International Institute of Information Technology Bangalore, Bangalore, Karnataka, India; ^2^Department of Electronics and Communication Engineering, Motilal Nehru National Institute of Technology Allahabad, Prayagraj, Uttar Pradesh, India; ^3^Department of Electronics and Computer Engineering, Thapar Institute of Engineering and Technology, Patiala, Punjab, India; ^4^Department of Electrical Engineering, Mathematics and Science, University of Gävle, Gävle, Sweden

**Keywords:** vision transformer, garbage classification, pyramid vision transformer, shifted window (Swin), garbage fullness detection

## Abstract

Efficient waste management is crucial for urban environments to maintain cleanliness, reduce environmental impact, and optimize resource allocation. Traditional waste collection systems often rely on scheduled pickups or manual inspections, leading to inefficient resource utilization and potential overflow issues. This paper presents a novel approach to automate the detection of garbage container fullness from images using machine learning techniques. More specifically, we explore three transformer-based architectures, namely, vision transformer, Swin transformer, and pyramid vision transformer to classify input images of garbage bins as clean or dirty. Our experimental results on the publicly available Clean dirty containers in Montevideo dataset suggest that transformer-based architectures are effective in garbage fullness detection. Moreover, a comparison with existing methods reveals that the proposed approach using the vision transformer surpasses the state-of-the-art, achieving a 96.74% accuracy in detecting garbage container fullness. In addition, the generalizability of the proposed approach is evaluated by testing the transformer-based classification frameworks on a synthetic image dataset generated using various generative AI models. The proposed approach achieved a highest test accuracy of 80% on this synthetic dataset, thereby highlighting its ability to generalize across different datasets. Synthetic dataset used in this work can be found at: https://www.kaggle.com/datasets/6df0652d2c4eb3b9f00043c40fba0afa0778b46d7c0685e212807c2f6967fe6f.

## 1 Introduction

Waste is one of the major factors in environmental pollution that the world faces. As the world's population increases, the production and consumption of industrial products will increase significantly and contribute to environmental waste (Catania and Ventura, 2014). To overcome this, waste management focuses on the proper and timely management of waste without causing any harm to human health and environmental wellbeing (Arthur et al., 2024). To facilitate the waste management process, temporary locations are allotted at various public spaces to dump and collect waste. One of the conventional methods is to dump waste in the containers placed at those temporary locations. The garbage trucks are then rotated to collect the waste from these containers (Laguna and Moncecchi, 2021). In this approach, the physical confirmation of waste collection personnel about the fullness of the containers is required by visiting its location (Lokhande and Pawar, 2016). Garbage collection from garbage containers is carried out first by manually checking the occupancy of each container, based on which the waste is then transferred to the garbage truck. Although it may appear to be a simple task, it is repetitive and mundane. It requires a significant amount of manual intervention to detect and evaluate a large number of containers throughout the city.

As humans we are prone to making mistakes, it what makes us human. Exhaustion, lack of motivation and carelessness can make this task a lot more difficult to be carried out properly. Some of these challenges are addressed by developing technologically advanced tools to manage, monitor, and control cleanliness in the city (Donati et al., 2019). Smart waste management is a technique many smart city projects aim to integrate. Various sensors are deployed to collect data from different parts of the city, and artificial intelligence (AI) techniques are utilized to analyze this data through the internet of things (IoT) network (Sohag and Podder, 2020). Such a smart system can be very useful for creating smart waste management solutions for smart cities. Previous attempts have been made to make trash bins for the university campuses (Longo et al., 2021). The trash bins are equipped with camera sensors to segregate the waste, and the IoT network controls the waste collection by monitoring the waste-filled in trash bins. Several research works focus on the classification of various waste items such as plastic, glass, tins, etc. (Sidharth et al., 2020; Ahmad et al., 2020; Nahiduzzaman et al., 2025). The work introduced by Oğuz and Ertuğrul (2023) focuses on the fullness of garbage collection containers. In Oğuz and Ertuğrul (2023), various deep-learning-based models are explored to classify whether garbage containers are fully or partially occupied. The proposed approach focuses on exploring various transformer-based models for the classification task.

The emergence of Vision Transformers (ViTs) marks a significant shift from conventional Convolutional Neural Networks (CNNs) in the field of deep learning (Dosovitskiy et al., 2021). Unlike CNNs, ViTs leverage the transformer architecture, originally introduced in natural language processing, to process images as sequences of patches. Through self-attention mechanisms, ViTs facilitate direct interactions among all patches, thereby enhancing global contextual understanding. This capability has led to state-of-the-art performance in a variety of computer vision tasks, including image classification. In this work, we demonstrate the effectiveness of ViTs in classifying images of garbage containers into clean and dirty categories, a problem domain that has received limited attention. We employ a pre-trained ViT model and fine-tune it on the publicly available Clean dirty containers in Montevideo (CDCM) dataset, containing labeled images of clean and dirty garbage bins. Additionally, we incorporate two state-of-the-art transformer-based pre-trained models, namely, the shifted window (Swin) transformer and the pyramid vision transformer (PVT), and fine-tune them for the same classification task. On the CDCM dataset, the ViT achieves the highest classification accuracy, outperforming both Swin transformer and PVT. As a further contribution and a key novelty of this work, we create a synthetic dataset using cutting-edge generative AI models, including GPT-4.0, Gemini, and Meta's frameworks GPT-4.0 (OpenAI, 2024; Gemini, 2025; AI, 2025). To assess the generalizability of the models on this dataset, we perform a cross-dataset analysis and evaluate all three fine-tuned transformers on the synthetic images. The satisfactory classification results affirm the robustness of the models across domains. Furthermore, we perform a comparative analysis with two existing deep CNN models and show that the ViT demonstrates superior performance. To enhance model interpretability, we conduct an analysis of intermediate layer outputs through heatmap visualizations and examine failure cases in detail.

## 2 Related works

In the context of standard dustbins, garbage is often observed to spill into the surrounding areas rather than being properly contained. This typically occurs due to several factors, such as the bin being full, the lid remaining closed or inaccessible, or poor disposal habits. While prior research has extensively explored various aspects of waste classification, such as dry vs. wet waste (Aravindaraman and Ranjana, 2019), metallic vs. non-metallic (Shamin et al., 2019), organic and recyclable landfill waste (Chhabra et al., 2024), biodegradable vs. non-biodegradable waste, and even hazardous waste such as poisonous gas emissions (Dubey et al., 2020), less attention has been given to the detection of bin fullness. In Sidharth et al. (2020), the authors focused on the classification of plastic, paper, cardboard, and metals. More recent studies have extended this scope to semantic segmentation and hybrid models. For example, In Qi et al. (2024), a semi-supervised approach was proposed for pixel-level segmentation of waste materials, enabling the classification of various objects in a given scene. Similarly, Lilhore et al. (2024) introduced a hybrid deep learning model combining CNNs with long short-term memory networks for categorizing waste into organic and recyclable types. Apart from this, multistage classification frameworks have also been explored to address the problem of recognizing various waste types. In Nahiduzzaman et al. (2025), the authors first performed binary classification between biodegradable and non-biodegradable waste, followed by a second stage of finer classification based on material characteristics. A real-time waste management system employing multiclass classification for various waste types, such as paper, glass, organic matter, plastic, e-waste, and metals, was proposed In Abozahhad and Abo-Zahhad (2025). Additionally, Tian et al. (2025) presented a classification approach tailored for orchard waste, introducing a specialized dataset containing items like fertilizer bags, pesticide containers, and cigarette butts.

Despite this progress, limited research efforts have focused specifically on detecting the fullness status of garbage containers. This is a crucial problem, especially in scenarios where resources are constrained, garbage collection is irregular, or manpower is insufficient. In this context, our work proposes a transformer-based framework to detect the fullness of garbage containers by analyzing the spillage of the waste around them. More importantly, we formulate this task as a binary classification problem, where the garbage bin images are categorized into either dirty or clean class. While previous studies (Oğuz and Ertuğrul, 2023) have addressed this problem using traditional CNNs or basic classification techniques, this work is among the first to evaluate and compare advanced transformer-based architectures for this task. These models leverage self-attention mechanisms, which enable them to capture long-range dependencies and contextual relationships across the entire image. In contrast, CNNs typically process local neighborhood information in a sequential manner, limiting their ability to model global spatial interactions. Moreover, unlike LSTM-based architectures (Lilhore et al., 2024), which are inherently sequential and often struggle with parallelization and long-range context, transformers can process entire sequences simultaneously, making them more efficient and effective for high-resolution image analysis.

## 3 Proposed approach

**Figure 1:**
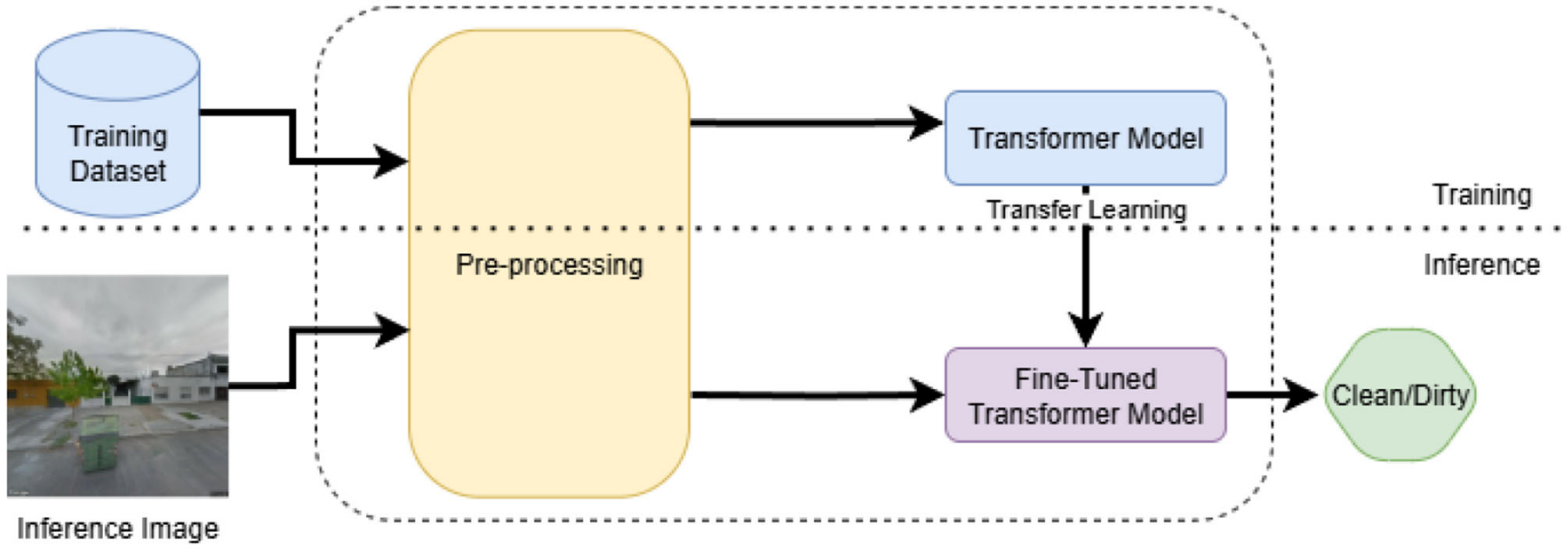
Block diagram of the proposed approach. During training, the pre-trained transformer model is fine-tuned using images of garbage bins and their corresponding labels. During inference, the fine-tuned model classifies whether a test image belongs to the class of clean or dirty.

In this section, we provide a detailed explanation of the various components and steps involved in the proposed transformer-based classification framework. A block diagram representation of the overall framework is shown in Figure 1, which outlines the complete workflow for detecting the fullness of garbage bins. The system takes images of garbage bins as input and classifies them into two distinct categories: clean (not yet full) or dirty (full or overflowing). The framework leverages the strength of pre-trained transformer models, specifically ViT, Swin transformer, and PVT, which are fine-tuned using a labeled dataset of garbage bin images from the publicly available CDCM dataset. The use of pre-trained models allows us to utilize the rich visual representations learned from large-scale datasets such as ImageNet, thereby accelerating convergence and improving performance, especially in limited data scenarios.

As illustrated in Figure 1, the first step in the pipeline involves a pre-processing block designed to prepare the images for model input. The pre-processing block resizes the images to match the input resolution required by each transformer architecture. In addition to resizing, we apply mean-standard deviation normalization to the pixel values of each image. This step ensures that the data distribution is consistent with the conditions under which the models were originally pre-trained, which helps in maintaining stable and effective learning. The pre-processed images, along with their corresponding binary labels, are then passed to the pre-trained transformer models. During the training phase, we adopt a transfer learning strategy (Zhu et al., 2011), wherein the base transformer weights are fine-tuned on the CDCM dataset while adapting the final classification layers to the specific task of garbage bin status prediction. This fine-tuning enables the models to learn task-specific features that enhance their ability to distinguish between clean and dirty bins. During inference, the trained model takes unseen test images as input and predicts whether these images belong to either clean or dirty classes based on the learned patterns.

### 3.1 Models

#### 3.1.1 Vision transformer (ViT)

**Figure 2:**
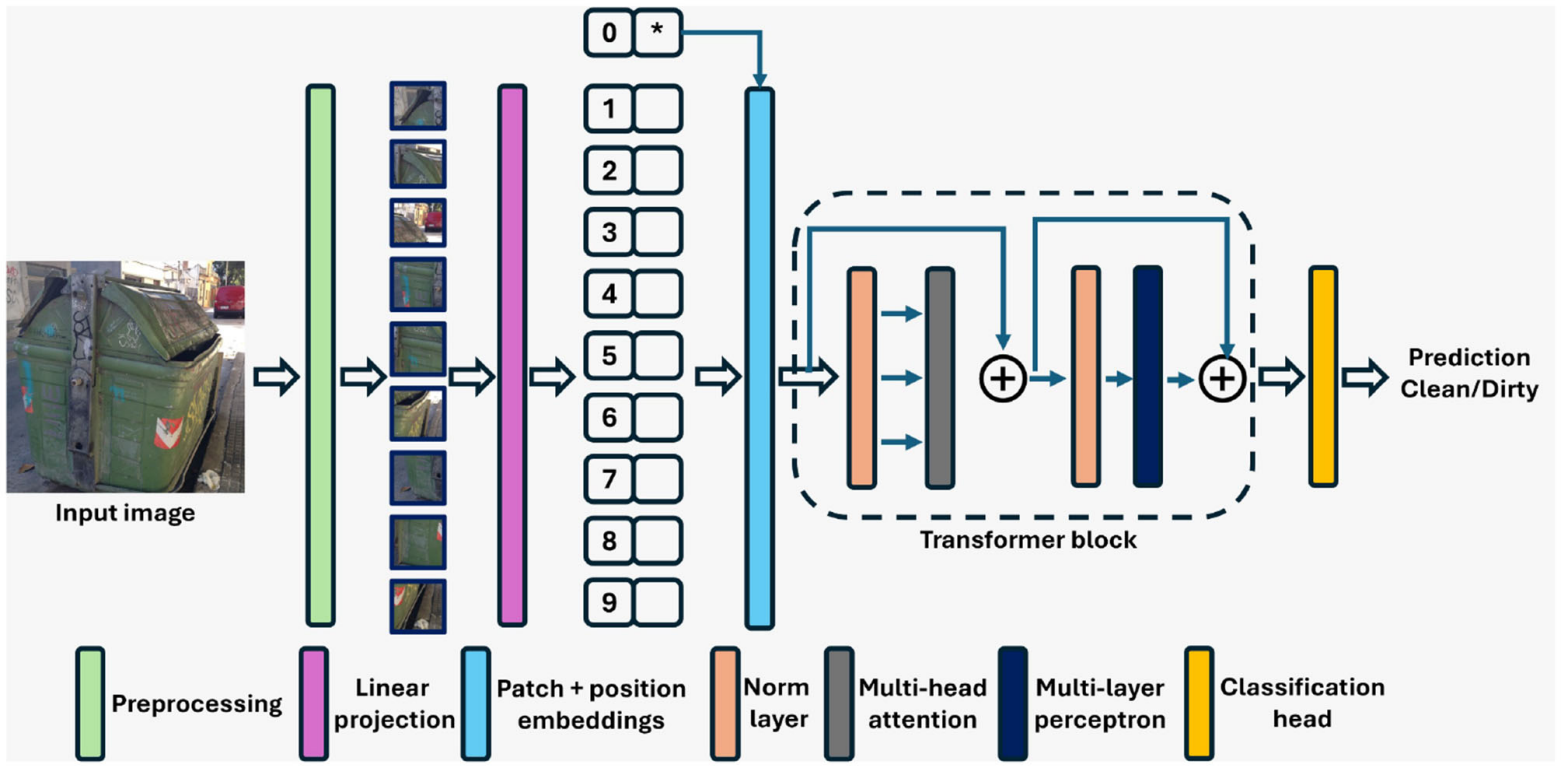
Architecture of the vision transformer (ViT) model utilized in the proposed classification framework. ^*^Refers to the extra learnable class embedding.

The ViT (Dosovitskiy et al., 2021) is a transformer encoder model trained using a large collection of images from the ImageNet-21k dataset (Ridnik et al., 2021) in a supervised manner. The block diagram of ViT is shown in Figure 2. Traditional image classification techniques rely mostly on CNNs, where convolution operators are used as filters on images. ViTs, on the other hand, utilize a novel approach. They use transformers to capture semantic information from the image to identify the relationship between different parts of an image. ViTs break down images into smaller patches, treating them as sequences similar to words in a sentence. These patches are then processed through multiple layers of self-attention mechanisms, allowing the model to understand the relationship and context between different parts of the images. There are three important layers in ViT's architecture: the pre-processing layer, transformer encoder, and classification head (Dosovitskiy et al., 2021). The pre-processing layer splits the image into smaller regions called patches, which are then flattened into vectors and fed through a linear layer. The linear layer reduces their dimensions and converts the vectors into lower-dimensional representations. Positional embeddings are then added to the patch vectors in order to encode the relative position of each patch within the image. The transformer encoder layer captures the relationship of each patch with all other patches in the sequence. Thus, it captures dependencies and interactions between different parts of the image (Dosovitskiy et al., 2021; Aaraki, 2024). It also consists of a FFN layer, which introduces non-linearity into the model. The classification head takes the transformer encoder output as its input and generates class probabilities for the image.

This unique architecture enables ViTs to capture global information and long-range dependencies within images effectively (Dosovitskiy et al., 2021; Aaraki, 2024). By utilizing ViT, we have leveraged their ability to analyze complex visual data and make accurate predictions about the occupancy status of garbage bins.

We employed a combination of cross-entropy loss and *L*2 regularization for our training process. Cross-entropy loss, commonly used in classification tasks, measures the difference between the predicted probabilities and the actual labels. This loss function penalizes incorrect classifications more severely, thereby encouraging the model to make accurate predictions. This method is also used for the Swin transformer and PVT.

#### 3.1.2 Shifted window transformer

**Figure 3:**
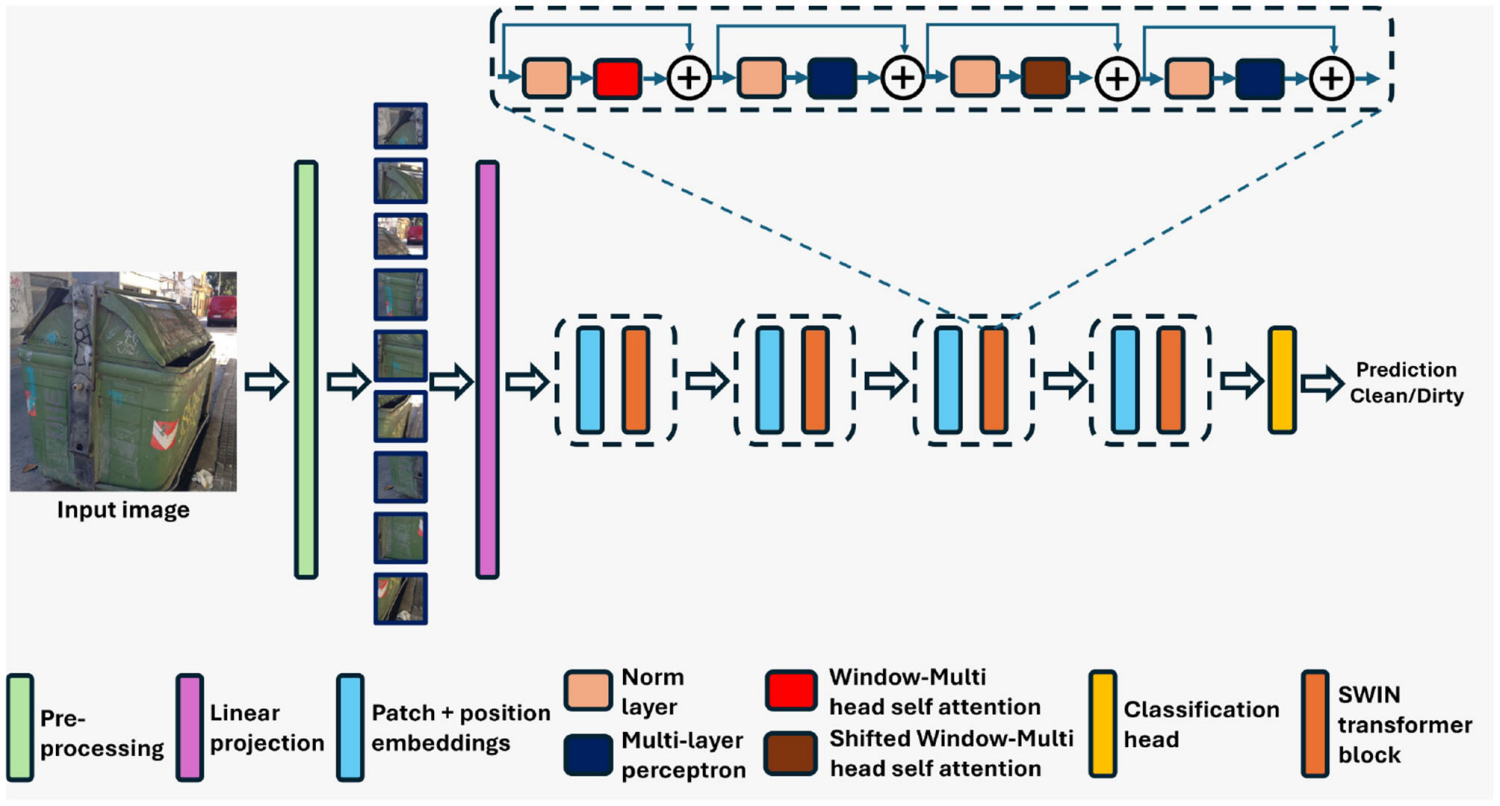
Architecture of the Swin transformer (Swin) utilized in the proposed classification framework.

The Swin transformer, similar to ViT, is a transformer-based deep architecture specifically designed for image processing tasks (Liu et al., 2021a,b). However, unlike ViT, the Swin transformer model used in this work was pre-trained on the ImageNet-1K dataset. Figure 3 presents the block diagram of the Swin model. Unlike traditional CNNs, Swin adopts a hierarchical processing strategy to effectively capture both local and global information within images. At its core, Swin operates by decomposing images into a series of patches, which are then processed through a series of transformer blocks. These transformer blocks work based on self-attention mechanism enabling the model to attend to the relationship between different patches at various hierarchical levels. This hierarchical processing allows this transformer model to capture both fine-grained details and global context within the image.

One distinguishing feature of Swin transformer is its hierarchical processing strategy, where patches are grouped into hierarchical stages. In each stage, patches are aggregated and processed to capture increasingly abstract features (Liu et al., 2021a,b). This hierarchical approach enables Swin transformer to effectively handle large images while maintaining computational efficiency. Furthermore, Swin transformer incorporates a multi-scale self-attention mechanism, which allows the model to capture both local and global dependencies within the image (Liu et al., 2021a,b). This mechanism enables Swin transformer to effectively model long-range dependencies while also maintaining spatial locality. In addition to the self-attention mechanism, Swin transformer also employs FFNs within each transformer block to introduce non-linearity into the model, further enhancing its representative capacity. Finally, similar to ViT, Swin transformer includes classification heads that take the output of the transformer blocks and produce predictions for the input image. These classification heads enable Swin transformer to perform tasks such as image classification (Huang et al., 2022), object detection (Gong et al., 2022), and semantic segmentation (Hatamizadeh et al., 2021).

#### 3.1.3 Pyramid vision transformer

**Figure 4:**
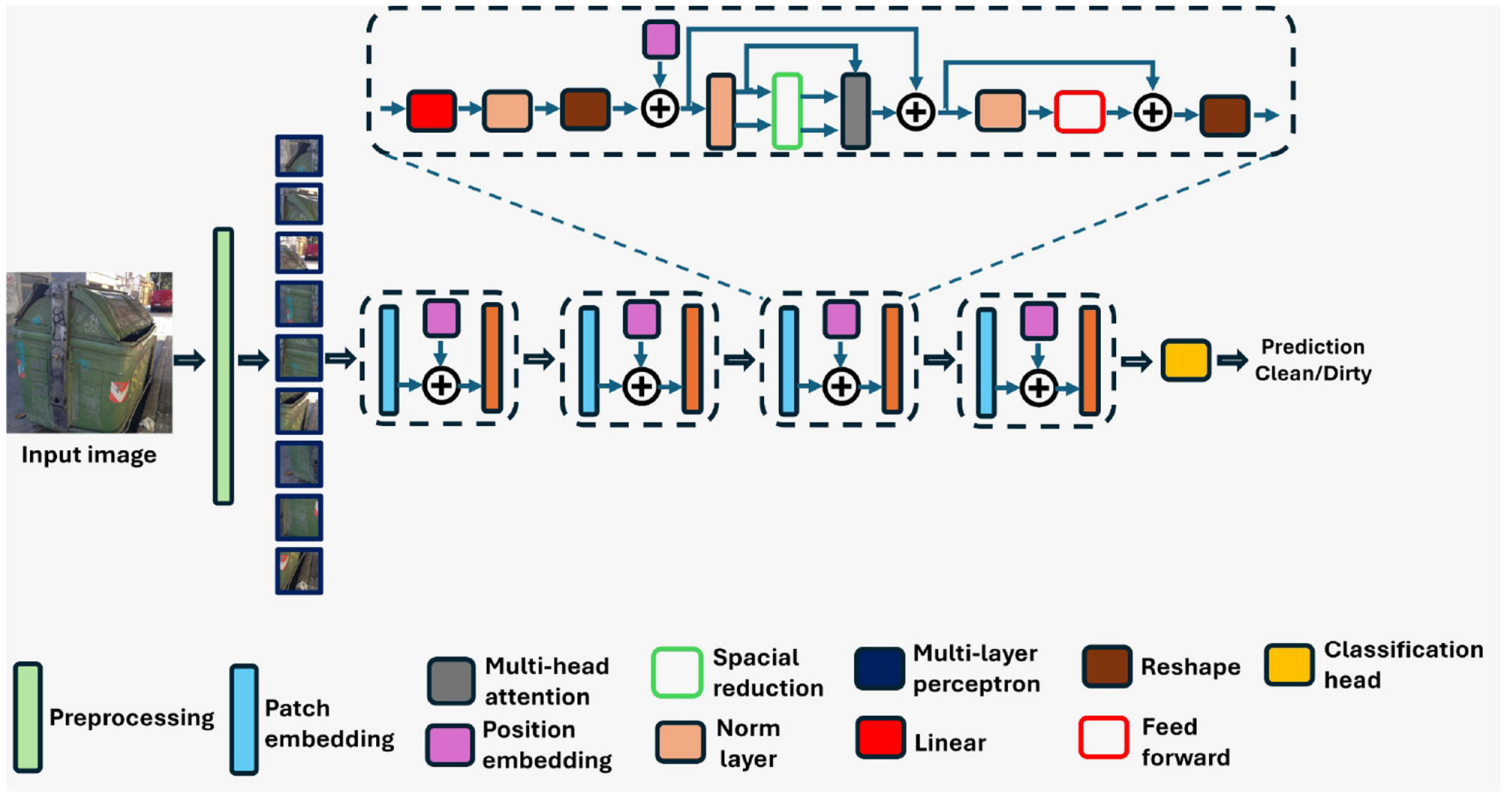
Architecture of the pyramid vision transformer (PVT) model utilized in the proposed classification framework.

The PVT (Wang et al., 2021) architecture combines transformer-based modeling with hierarchical feature processing, making it well-suited for image understanding tasks (Wang et al., 2021).^1^ The PVT model used in this study was pre-trained on the ImageNet-1K dataset. Figure 4 shows the architecture of PVT. Unlike traditional CNNs, PVT leverages transformers' strengths in capturing long-range dependencies and global context in images (Wang et al., 2021) (see text footnote ^1^). It begins by decomposing images into patches, akin to tokens in natural language processing tasks. However, PVT takes a step further by introducing a pyramid structure to capture features at multiple scales. This pyramid structure consists of multiple levels, each processing patches at different spatial resolutions. This enables the PVT to efficiently capture both local details and global context across the entire image (Wang et al., 2021) (see text footnote ^1^). Within each level of the pyramid structure, PVT employs transformer blocks to process the patches. These transformer blocks utilize self-attention mechanisms to model dependencies between patches, allowing the model to understand relationships and context within the image. Additionally, PVT introduces positional embeddings to encode the spatial information of patches, ensuring that the model can distinguish between different regions of the image.

One key aspect of PVT is its ability to aggregate information across different levels of the pyramid structure. By incorporating features from multiple scales, PVT can effectively capture hierarchical representations of the input image, leading to richer and more informative representations (Wang et al., 2021) (see text footnote ^1^). Furthermore, PVT includes classification heads that take the aggregated features from the pyramid structure and produce predictions for the input image.

In the proposed work, we selected these three transformer-based architectures based on their proven effectiveness and representativeness of different architectural paradigms in the vision transformer family. These models are widely cited in literature and offer a comprehensive comparison across flat (ViT), hierarchical (PVT), and shifted-window-based (Swin) transformer designs, allowing us to explore their relative strengths for the task of garbage bin fullness detection. ViT employs a flat structure and processes images as a sequence of non-overlapping patches using global self-attention (Dosovitskiy et al., 2021). Its simplicity allows for faster inference, but it lacks inherent locality and multi-scale feature extraction, which may reduce performance on complex scenes without sufficient data or inductive biases. Swin transformer introduces a hierarchical design with shifted window-based attention, enabling local context modeling and efficient computation (Liu et al., 2021b). It balances accuracy and computational cost well but can have slower inference due to window shifting and patch merging overhead. PVT, on the other hand, adopts a pyramid structure, with spatial reduction attention to reduce complexity while preserving performance across scales (Wang et al., 2021). It offers lower parameter count and better performance for dense prediction tasks but may be slightly less effective than ViT in capturing global dependencies for simpler classification problems.

## 4 Dataset and experiments

In this section, we briefly describe the dataset employed in this work. Apart from the dataset, we present the experimental protocols and performance measures utilized in the proposed approach.

### 4.1 Dataset

In this study, we have employed the Clean dirty containers in Montevideo (CDCM) dataset (Laguna, 2021). This dataset is created by Laguna and Moncecchi (2021) to automatically notify the authorities for collecting garbage accumulated during the pandemic, disturbing the environment. Platforms such as Google Street View, local complaint platforms of municipalities, and search engines were used while creating the dataset. There are 3,414 images in the dataset, of which 1,806 and 1,608 images belong to clean and dirty classes, respectively. Additionally, the dataset has already been divided into train and test datasets. The train and test datasets consist of 2,217 and 1,197 images respectively.

**Figure 5:**
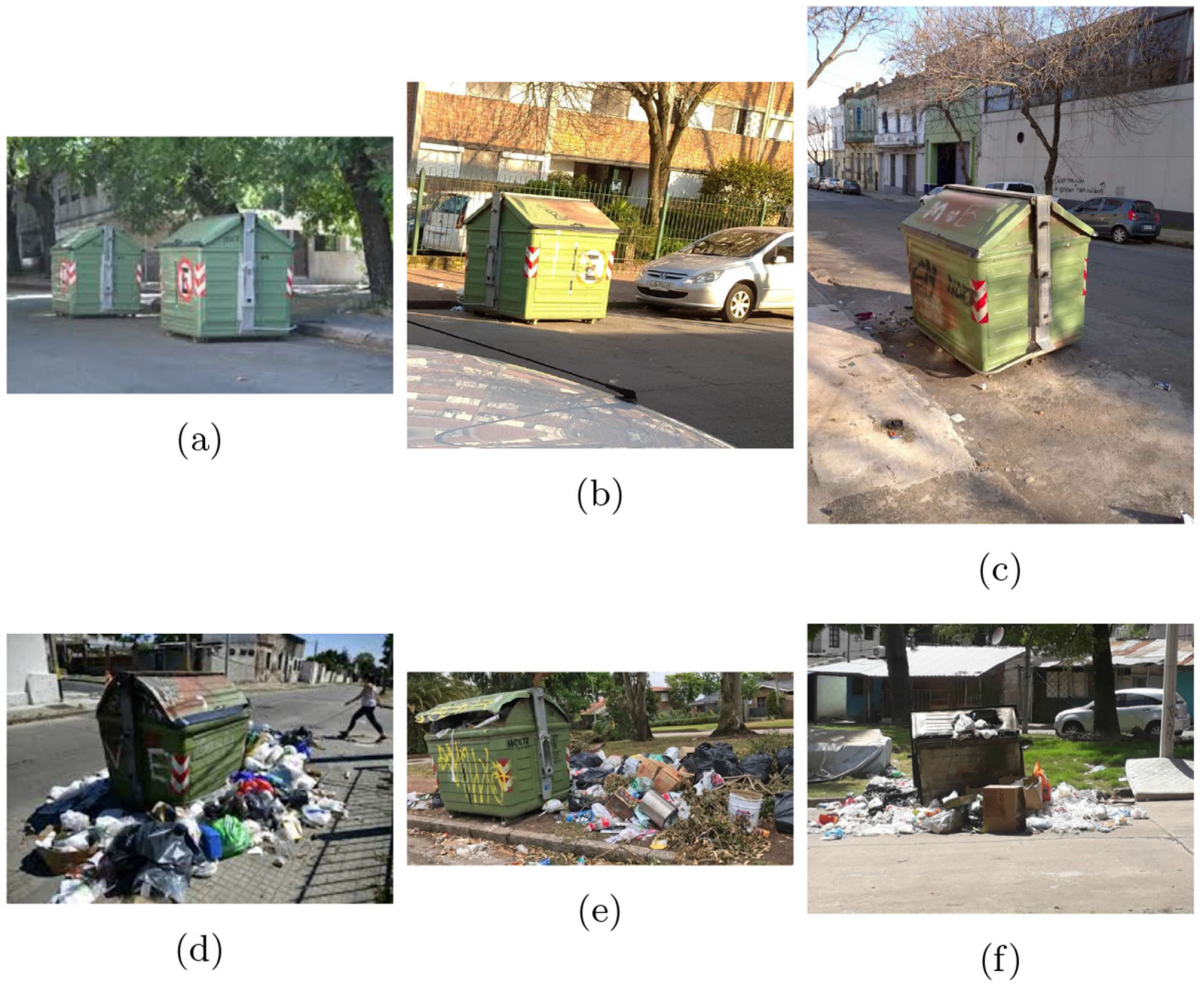
Sample images from the Clean dirty containers in Montevideo (CDCM) dataset. **(a–c)** Belong to clean class and **(d–f)** belong to dirty class.

In the CDCM dataset, images labeled as “dirty” typically depict garbage bins that are fully occupied, often with additional piles of waste placed beside the containers. In contrast, images labeled as “clean” generally show bins that are not yet full. The sample images of the dataset are shown in Figure 5, where images from the dirty class clearly show overflowing bins and surrounding waste, indicating the need for prompt intervention. The images in this dataset are originally available in varying sizes and resolutions, ranging approximately from 600 × 600 to 10, 338 × 3, 168 pixels, as they were captured at different locations and over different time periods. Therefore, resizing and normalization of the data was important. The images were all converted to JPG and then resized as per the requirements of individual models. For ViT and PVT, the images were resized to have 224 × 224 pixels, whereas for Swin transformer, they were resized to the dimensions of 256 × 256. The dataset is already split into training and testing datasets. The images were then loaded into data loaders with a batch size of 16.

### 4.2 Experimental protocol

For the training process, we adopted a transfer learning approach utilizing pre-trained transformer models. The ImageNet pre-trained weights for ViT, SWIN transformer, and PVT were obtained from Aaraki (2024), Microsoft (2024); Liu et al. (2021a), and see text footnote ^1^, respectively. There exists a certain degree of visual similarity and feature correlation between the ImageNet dataset and the CDCM dataset used in this study. Pre-training on a large and diverse dataset like ImageNet enables the model to learn robust and generalizable visual features, which can significantly enhance performance when fine-tuned on a domain-specific task such as garbage bin cleanliness classification. We have chosen particular model versions based on different factors such as window size and the dataset on which they were previously trained. These models have already been trained for image classification. We have fine-tuned the transformer models for the task of garbage bin fullness classification using the images from the CDCM dataset. For this purpose, layer freezing was used and only the last layer i.e., the classifier head, was fine-tuned on the new dataset with classes: “clean” and “dirty.” Freezing the layers except the classification head allows us to retain information and patterns learned by the model on the images it was initially trained on, while also allowing the model to learn new features for the required task (Goutam et al., 2020).

The training was carried out using Adam optimizer and binary cross-entropy loss function, defined in Equation 1. The initial learning rate (LR) for each model is kept at 10^−5^. The LR was then decremented during the training stage using cosine annealing. Cosine annealing is a LR scheduling technique which dynamically adjusts the LR during training by gradually decreasing it in a cosine-shaped manner (Gotmare et al., 2018). Decreasing the LR during the training process helps in overcoming the issue of flat plateau in optimizing the loss function and converge more smoothly near minimal loss point (LeCun et al., 1988).


(1) $$\begin{array}{l} BCE=-\left\{y\log\left(p\right)+\left(1-y\right)\log\left(1-p\right)\right\} \end{array}$$

Where, *y* is a binary indicator whose value is 1 in case of correct prediction i.e., when the observation *o* is correctly classified into class *c* and 0 otherwise. *p* is the probability of the observation *o* belonging to class *c*.

Given the computational demands of transformer-based architectures, model training was performed using high-performance graphics processing units (GPUs). Specifically, we utilized the freely available NVIDIA P100 GPU on the Kaggle platform.^2^ The experiments are performed on a Windows 8-GB RAM system equipped with an Intel Core i5 CPU. The implementation was carried out in Python using the PyTorch deep learning framework. To prevent overfitting and improve generalization to unseen data, we incorporated multiple regularization strategies. First, we applied *L*_2_ regularization by adding a penalty term to the loss function, discouraging the model from learning excessively large weights (Drucker and Le Cun, 1992). Additionally, we employed dropout and early stopping to reduce the risk of overfitting.

Dropout was applied in the final classification layer of each model, where a fraction of input units was randomly set to zero during training. This technique reduces co-adaptation among neurons and encourages the model to learn more robust and generalized features by training on varying subsets of the data. Specifically, dropout probabilities of 0.4, 0.2, and 0.4 were used for the ViT, Swin Transformer, and PVT models, respectively. These values were determined experimentally based on optimal classification performance during validation. Dropout was applied in the final classification layer of each model, where a fraction of input units was randomly set to zero during training. This technique reduces co-adaptation among neurons and encourages the model to learn more robust and generalized features by training on varying subsets of the data. Specifically, dropout probabilities of 0.4, 0.2, and 0.4 were used for the ViT, Swin Transformer, and PVT models, respectively. These values were determined experimentally based on optimal classification performance during validation. Early stopping was also implemented to halt training once performance on the validation set began to degrade or plateau, thereby preventing unnecessary training epochs and further reducing the risk of overfitting. Each model was initially trained for up to 20 epochs. Table 1 shows the summary of the hyperparameters used in our experiments.

**Table 1:** Summary of the hyperparameters used in the experiments.

**Model configuration**	**ViT**	**Swin transformer**	**PVT**
Input dimension	224 × 224	256 × 256	224 × 224
Pre-trained weights	ImageNet-21k	ImageNet-1k	ImageNet-1k
Number of encoder layers	12	24	8
Number of attention heads	12	Range: [4–32]	Range: [1–8]
Patch sizes	16 × 16	4 × 4	Range: [(4 × 4)–(32 × 32)]
**Training hyperparameters**	**ViT**	**Swin transformer**	**PVT**
Train-test split	As provided in Laguna and Moncecchi (2021)	As provided in Laguna and Moncecchi (2021)	As provided in Laguna and Moncecchi (2021)
Dropout rate	0.4	0.2	0.4
Loss function	*BCE*	*BCE*	*BCE*
Regularization	*L*_2_	*L*_2_	*L*_2_
Epochs	20	20	20
Initial learning rate	10^−5^	10^−5^	10^−5^
Optimizer	Adam	Adam	Adam
Batch size	16	16	16

### 4.3 Performance metrics

We have employed performance metrics such as accuracy, loss, and F1-score to measure the classification performance of the transformer models on the test set of the dataset. We have plotted receiver operating characteristics (ROC) curves corresponding to all three transformer models. The area under the ROC curve (AUC) is also computed as an important performance metric.

F1-score is calculated as


(2) $$\begin{array}{l} F1-score=2\times \frac{precision\times recall}{precision+recall}, \end{array}$$

where precision and recall are given as:


(3) $$\begin{array}{l} Precision=\frac{TruePositives}{TruePositives+FalsePositives}, \end{array}$$

(4) $$\begin{array}{l} Recall=\frac{TruePositives}{TruePositives+FalseNegatives}. \end{array}$$

The *F*1−*score* ranges from 0 to 1, where a score of 1 indicates perfect precision and recall, and a score of 0 indicates poor performance in either precision or recall. F1-score is useful when there is an imbalance between the number of positive and negative instances in the dataset, as it considers both false positives and false negatives. Additionally, we computed stratified performance metrics of all three transformer-based classifiers by calculating the class-wise precision and recall metrics as follows:


(5) $$\begin{array}{l} Precision_{C}=\frac{TruePositives_{C}}{TruePositives_{C}+FalsePositives_{C}}, \end{array}$$

(6) $$\begin{array}{l} Recall_{C}=\frac{TruePositives_{C}}{TruePositives_{C}+FalseNegatives_{C}}. \end{array}$$

Here, *Precision*_*C*_ and *Recall*_*C*_ correspond to the precision and recall metrics for class *C*∈{0, 1}. We have also added the confusion matrices for the models, which clearly show the actual and predicted parameters. The area under the ROC curve allows us to visualize how sensitivity and specificity are traded off. Greater discrimination between positive and negative instances is typically exhibited by a model with a higher AUC score.

## 5 Results and discussion

In the proposed approach, we employed the transfer learning method to fine-tune the pre-trained ViT, Swin transformer, and PVT models for the task of garbage bin fullness detection. Table 2 reports the classification performance of these models in terms of accuracy, loss, F1-score, and AUC. The results clearly demonstrate the effectiveness of the proposed method in classifying images into clean or dirty categories. From Table 2, it can be observed that the ViT-based classification framework outperforms both the Swin Transformer and PVT-based models. Specifically, the ViT model achieves the highest accuracy of 96.74%, F1-score of 96.60%, and AUC of 0.97, indicating superior overall performance. We also observe that the ViT-based classifier achieves higher precision for Class 1 (dirty bins) and higher recall for Class 0 (clean bins), signifying that this model is particularly effective at correctly identifying clean bins, thereby minimizing false positives. Furthermore, we performed statistical significance analysis using the confidence interval (CI) method. The results corresponding to this analysis are presented in Table 2. The ViT-based architecture achieved an accuracy of 96.74%, and at a 95% confidence level, its true accuracy is estimated to lie between 95.74% and 97.66%. This narrow confidence interval reflects a high degree of precision in the model's performance estimate, further validating its robustness and reliability.

**Table 2:** Performance of the transformer-based classification frameworks on the CDCM dataset.

**Model**	**Performance metrics**					**Class-wise metrics (Class 0/1)**	
	**Accuracy (%)**	**Confidence interval**	**Loss**	**F1-score (%)**	**AUC**	**Precision (%)**	**Recall (%)**
ViT	96.74	95%, [0.9574–0.9766]	0.121	96.60	0.97	95.00/99.00	99.00/95.00
Swin	95.74	95%, [0.9457–0.9691]	0.117	95.60	0.96	95.00/97.00	97.00/95.00
PVT	95.49	95%, [0.9432–0.9666]	0.131	95.50	0.95	96.00/95.00	95.00/96.00

**Figure 6:**
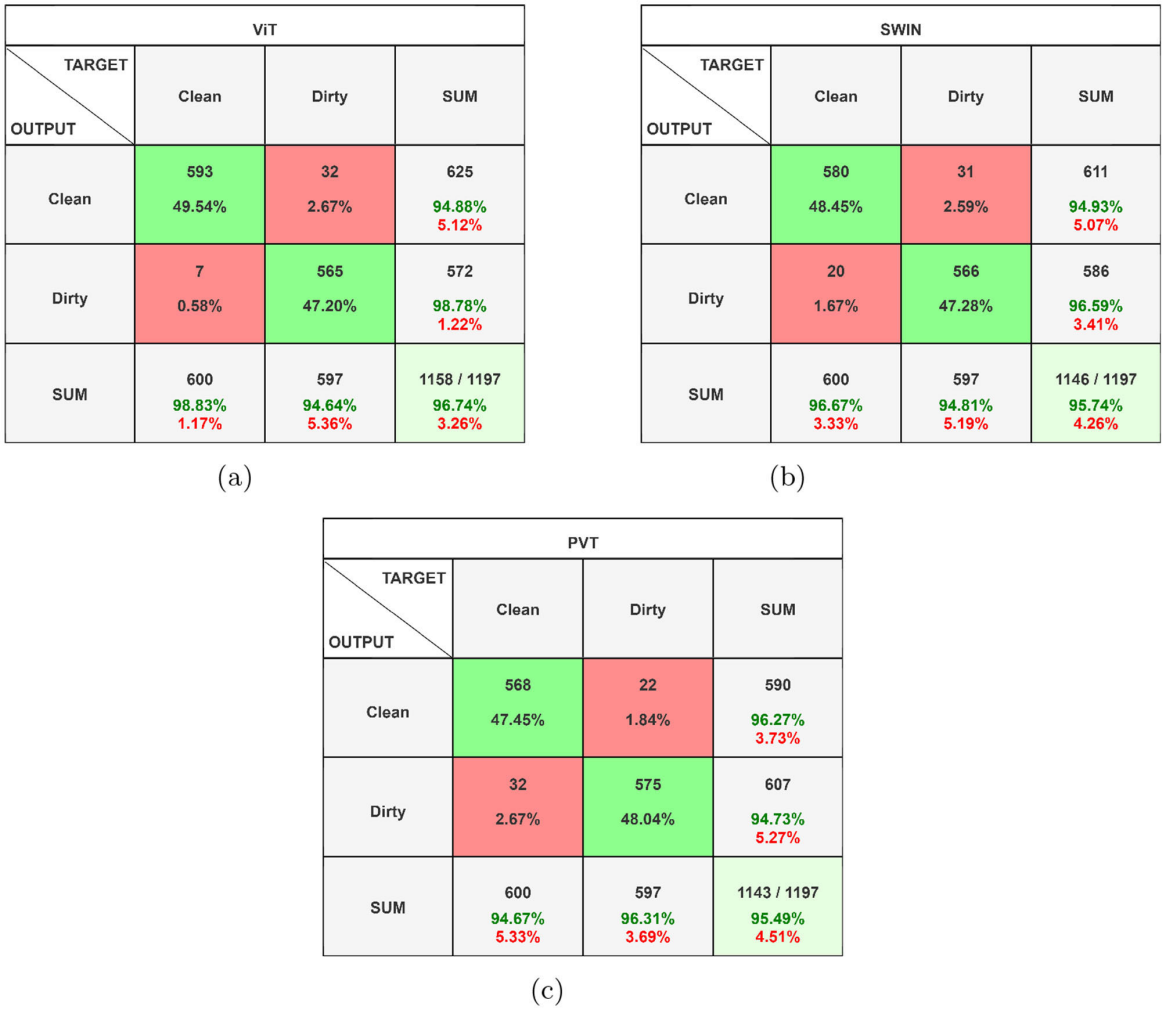
Confusion matrices corresponding to **(a)** ViT, **(b)** Swin transformer, and **(c)** PVT-based classifiers (best viewed in color).

**Figure 7:**
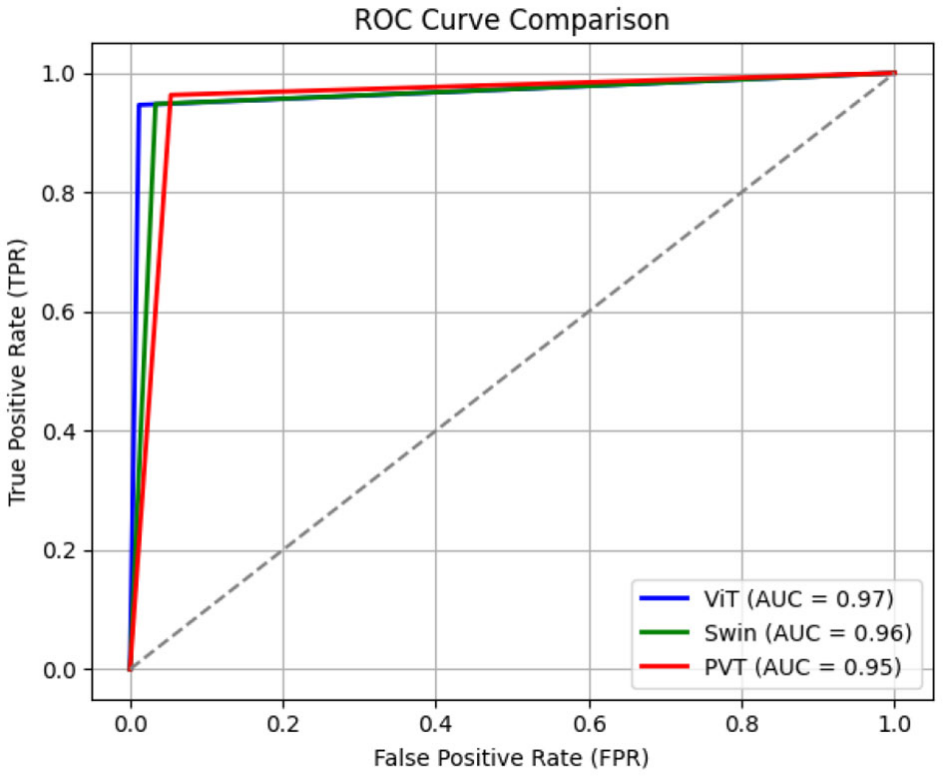
Receiver operating characteristic (ROC) curves corresponding to ViT-, Swin transformer-, and PVT-based classifiers.

**Figure 8:**
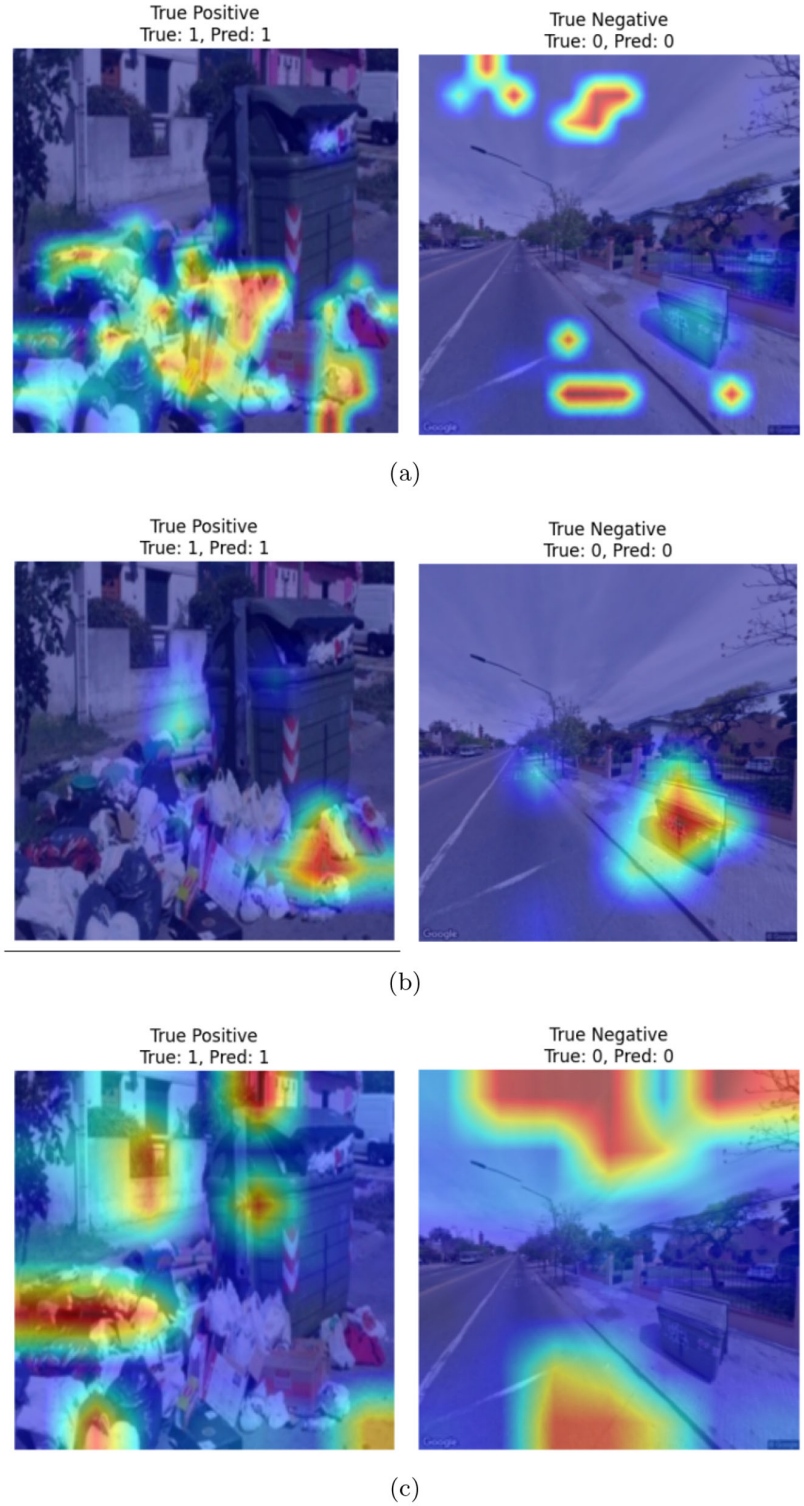
Heatmap visualization of correct predictions corresponding to **(a)** ViT-, **(b)** Swin transformer-, and **(c)** PVT-based classifiers.

Figure 6 shows the confusion matrices corresponding to the ViT-, Swin Transformer-, and PVT-based classification frameworks. As evident from the figure, all three models achieve comparable true positive and true negative rates. However, the ViT-based framework demonstrates a significantly lower false negative rate, misclassifying only 7 out of 600 clean garbage bins as dirty. The ROC curves for all three models are shown in Figure 7, where the AUC for the ViT model is observed to be higher than those of the Swin transformer and PVT, indicating better overall classification performance. Furthermore, Figure 8 shows the intermediate layer heatmap visualizations (Selvaraju et al., 2017) of these models corresponding to true predictions. The left column represents true positive cases, while the right column shows true negatives. These visualizations indicate that the models are able to extract and utilize discriminative features to distinguish between clean and dirty bins. Notably, the ViT model demonstrates superior capability in attending to relevant spatial regions, leading to more accurate classification decisions.

Additionally, we compared the computational complexity of all three models based on the number of parameters and the number of Giga multiply-accumulate (GMAC) operations. As shown in Table 3, the PVT model exhibits significantly fewer parameters and lower GMAC operations compared to the ViT and Swin Transformer models. However, due to its simpler and non-hierarchical architecture, ViT achieves faster inference time per image (0.94 ms), outperforming both PVT (2.32 ms), and Swin Transformer (5.33 ms) in terms of speed.

**Table 3:** Comparison of computational complexity of all three transformer-based models.

**Model**	**# Parameters**	**# GMAC operations**	**Inference time**
ViT	85.6 million	16.8 billion	0.94 ms/image
Swin transformer	87.9 million	19.9 billion	5.33 ms/image
PVT	43.8 million	6.4 billion	2.32 ms/image

### 5.1 Performance comparison

Table 4 presents a performance comparison of the proposed approach with the existing approach in this domain. Given the limited work explored in this field, we compare our proposed approach with the only available existing method (Oğuz and Ertuğrul, 2023). From Table 4, it can be observed that the proposed approach outperforms the existing method in detecting the fullness of the garbage container. More specifically, all transformer-based models employed in our work have consistently achieved higher accuracy with comparatively lower loss than the CNN models used in Oğuz and Ertuğrul (2023).

**Table 4:** Performance comparison of the proposed approach with existing work.

**Model**		**Performance metrics**	
		**Accuracy (%)**	**Loss**
Oğuz and Ertuğrul (2023)	DenseNet-169	90.35	0.425
	EfficientNet-B3	90.18	0.515
	MobileNetV3-Large	88.89	0.424
	VGG19-Bn	94.93	0.382
Our results	ViT	**96.74**	**0.121**
	Swin transformer	95.74	0.117
	PVT	95.49	0.131

Bold values indicates best achieved among other values.

To further evaluate the effectiveness of the transformer-based models, we performed experiments using conventional CNN models for the task of garbage bin fullness detection. Specifically, we employed two popular CNN architectures, ResNet50 (He et al., 2016) and VGG-16 (Simonyan and Zisserman, 2014), and fine-tuned their weights using the CDCM dataset. Both ResNet50 and VGG-16 were pre-trained on the ImageNet-1k dataset with an input resolution of 224 × 224. During fine-tuning, we replaced the original classification layer with a new output layer consisting of two neurons to accommodate the binary classification task. The models were fine-tuned using the Adam optimizer and binary cross-entropy loss, with a weight decay of 10^−3^. A learning rate of 10^−5^ was employed, along with the cosine annealing learning rate scheduling technique to facilitate efficient convergence. The transformer models consistently outperformed the conventional CNN models on the same dataset. As reported in Table 5, ResNet50 achieved a classification accuracy of 72.68%, while VGG-16 reached 79.45%, both significantly lower than the accuracy obtained by the transformer-based models.

**Table 5:** Performance comparison of the proposed approach with standard convolutional neural networks (CNNs).

**Model**	**Performance metrics**			
	**Accuracy (%)**	**Loss**	**F1-score (%)**	**AUC**
ResNet50	72.68	0.581	71.50	0.73
VGG-16	79.45	0.449	79.10	0.79
ViT	**96.74**	**0.121**	**96.60**	**0.97**

Bold values indicates best achieved among other values.

### 5.2 Cross-dataset analysis

**Figure 9:**
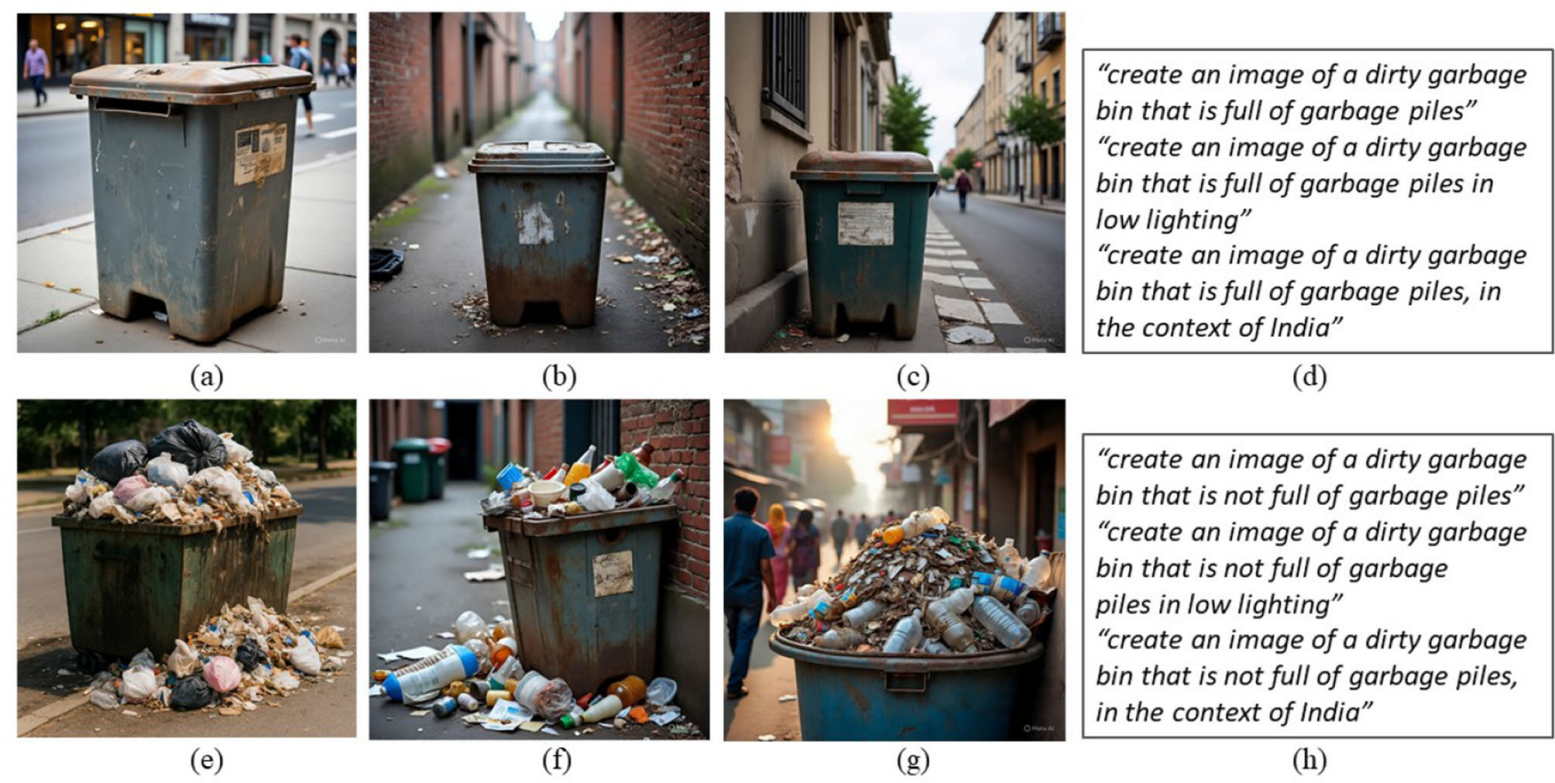
Sample synthetic images generated using various generative AI models and the corresponding meta prompts. **(a–c)** Sample images from the clean class, **(e–g)** Sample images from the dirty class, **(d)** set of meta prompts to generate images from the clean class, and **(h)** set of meta prompts to generate images from the dirty class.

To evaluate the generalizability of the transformer-based models under diverse imaging conditions, we conducted a cross-dataset analysis using a balanced dataset of synthetic images comprising empty and full dustbins. We generated these images using state-of-the-art multimodal large language models, including GPT-4.0 (OpenAI, 2024), Gemini (Gemini, 2025), and Meta's (AI, 2025) generative frameworks. The prompts used for image generation are presented in Figures 9d, h, and were carefully designed to reflect a wide range of real-world lighting conditions and regional characteristics, as illustrated in Figures 9a–c, e–g. The synthetic dataset^3^ consists of 50 images in total, with 25 samples per class. Table 6 presents the classification performance of the transformer-based models when evaluated on this synthetic dataset. All three models, which were fine-tuned on the publicly available CDCM dataset, achieved satisfactory classification results, demonstrating their ability to generalize across domains. Interestingly, the PVT- and Swin Transformer-based classifiers outperformed the ViT model in this cross-dataset evaluation. This may be attributed to the focused composition of the synthetic images, which predominantly emphasize the garbage bin itself, unlike the more cluttered and varied scenes found in the CDCM dataset (see Figure 5).

**Table 6:** Effect of cross-dataset analysis: performance of the transformer-based classification frameworks fine-tuned on the clean dirty containers in montevideo (CDCM) dataset, evaluated on the synthetic dataset.

**Model**	**Performance metrics**		
	**Accuracy (%)**	**Loss**	**F1-score (%)**
ViT	70.00	0.615	67.00
Swin transformer	80.00	0.350	79.00
PVT	80.00	0.587	79.00

The proposed approach has significant potential for deployment across various real-world platforms aimed at enhancing urban sanitation infrastructure. Applications include integration into smart city waste management systems, where real-time monitoring of bin cleanliness can optimize collection schedules and reduce operational costs. Additionally, embedding the detection model into IoT-enabled smart bins or mobile applications can facilitate on-site decision-making for sanitation workers.

## 6 Ablation studies

We conducted a series of ablation studies to evaluate the effectiveness of the proposed ViT model in classifying garbage bin images into empty and full categories. To this end, we compared the classification performance of the model, fine-tuned using the CDCM dataset, with its counterpart, where the training is performed from scratch. In the latter setting, the model weights were initialized randomly, in contrast to the fine-tuned model, which leveraged pre-trained weights from ImageNet. The results of this comparison, reported in Table 7, show a significant improvement in performance due to fine-tuning, where the classification accuracy increased from 68.00% to 96.74% and the loss decreased from 0.617 to 0.121. These findings underscore the effectiveness of transfer learning in this context, likely due to the visual similarity and feature-level correlations between the ImageNet dataset and the CDCM dataset.

**Table 7:** Effect of transfer learning on performance of the vision transformer (ViT) model.

**Model**	**Performance metrics**			
	**Accuracy (%)**	**Loss**	**F1-score (%)**	**AUC**
ViT (Training from scratch)	68.00	0.617	64.37	0.68
ViT (Fine-tuning)	**96.74**	**0.121**	**96.60**	**0.97**

Bold values indicates best achieved among other values.

Additionally, to study the effect of the number of encoder layers in the ViT model, we varied the number of encoder layers and fine-tuned its parameters using the protocol outlined in Section 3.1. Table 8 summarizes the performance of the proposed ViT-based framework and its ablated variants in terms of accuracy, loss, F1-score, and AUC. The results indicate that reducing the number of encoder layers leads to a decline in classification accuracy and F1-score, along with an increase in the loss value, highlighting the importance of architectural depth in achieving optimal performance.

**Table 8:** Effect of varying the number of encoder layers on performance of the vision transformer (ViT) model.

**Number of layers**	**Accuracy (%)**	**Loss**	**F1-score (%)**	**AUC**
12 (Default)	**96.74**	**0.121**	**96.60**	**0.97**
10	94.90	0.223	94.50	0.95
8	95.66	0.144	95.30	0.95
6	93.90	0.167	93.90	0.94

Bold values indicates best achieved among other values.

To evaluate the impact of the number of attention heads in the ViT architecture, we conducted additional experiments by varying this hyperparameter. As discussed in Section 3.1, we employed a pre-trained ViT model and fine-tuned its weights on the CDCM dataset for the classification task. It is worth noting that the default number of attention heads in a pre-trained ViT is 12. The default configuration of the pre-trained ViT includes 12 attention heads. To investigate the effect of modifying this setting, we trained ablated versions of ViT from scratch using different numbers of attention heads. For consistency, we also trained a ViT with the default 12-head configuration from scratch. Table 9 shows the performance comparison across these configurations. The results demonstrate that the ViT with the default number of attention heads consistently outperforms the modified versions, even when trained from scratch. This highlights the critical role of attention head configuration in maintaining the model's representational capacity and classification performance for garbage bin fullness detection.

**Table 9:** Effect of varying the number of attention heads on performance of the vision transformer (ViT) model.

**Number of heads**	**Performance metrics**			
	**Accuracy (%)**	**Loss**	**F1-score (%)**	**AUC**
4	66.75	0.618	63.41	0.67
8	67.00	0.626	66.77	0.67
12 (Default)	**68.00**	**0.617**	**64.37**	**0.68**
16	67.92	0.700	61.36	0.68

Bold values indicates best achieved among other values.

## 7 Discussion on failure cases

**Figure 10:**
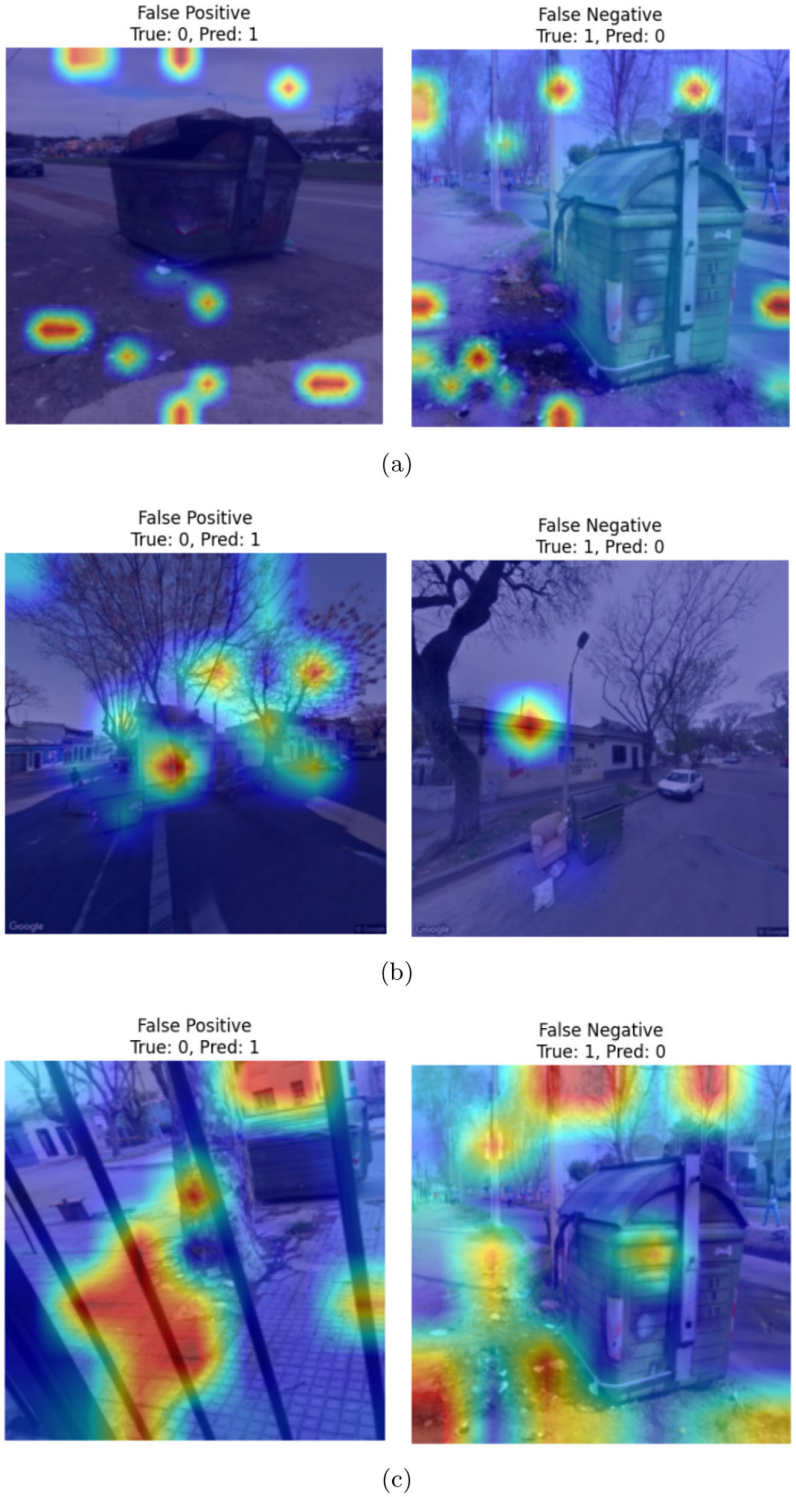
Examples of failure cases, superimposed with heatmaps, corresponding to **(a)** ViT-, **(b)** Swin transformer-, and **(c)** PVT-based classifiers.

Figure 10 illustrates example images showing failure cases from the CDCM dataset considered in our experiments. The first column displays false positives, where images from the clean category were incorrectly classified as dirty. The second column shows false negatives, where images from the dirty category were misclassified as clean. Heatmaps are superimposed on each image to visualize the highlighted regions that may have contributed to the misclassification. As observed, false positives primarily occur in scenarios where the garbage bin is partially occluded, making it difficult for the model to assess its actual status. In contrast, false negatives are typically associated with images where the visible amount of trash is relatively low, leading the model to misinterpret the bin as clean. Overall, the heatmaps indicate that in both types of errors, the models tend to focus more on spurious features such as background regions rather than the garbage bin itself, which likely contributes to the misclassification.

## 8 Conclusion

Automated detection of the fullness of Garbage containers offers many advantages like reduced manual checks and efficient waste collection which in turn lead to reduced costs. In this paper, we developed an automated approach for detecting the fullness of garbage containers using vision transformers. Our approach has achieved the highest accuracy of 96.74% in detecting garbage fullness using ViT. Our experimental results on a publicly available CDCM dataset suggest that the proposed approach is effective in detecting garbage fullness, and it outperformed existing approaches. Additionally, the satisfactory classification performance on a synthetic dataset, reflecting various real-world lighting conditions, highlights the potential of this approach in practical applications such as smart city and sanitation. As part of future work, we plan to explore novel architectures to reduce the false predictions further. Additionally, we intend to leverage state-of-the-art generative models to create high-quality synthetic data, which can augment the existing dataset and enhance the robustness and generalization capabilities of the proposed models.

## Data Availability

The original contributions presented in the study are included in the article/supplementary material, further inquiries can be directed to the corresponding author. The synthetic dataset containing 25 images per class (Fullness and Non-Fullness) with varying resolutions is available at: https://www.kaggle.com/datasets/6df0652d2c4eb3b9f00043c40fba0afa0778b46d7c0685e212807c2f6967fe6f.

## Footnotes

^1^Google colab. (2024). Available online at: https://huggingface.co/Zetatech/pvt-medium-224.

^2^Kaggle code. (2024). Available online at: https://www.kaggle.com/code.

^3^Synthetic dataset for garbage bin fullness detection. (2025). Available online at: https://kaggle.com/datasets/6df0652d2c4eb3b9f00043c40fba0afa0778b46d7c0685e212807c2f6967fe6f (Accessed July 24, 2025).
